# Vertical distribution and migration of microplastics in soils from Fars Province, Southwest Iran

**DOI:** 10.1371/journal.pone.0333572

**Published:** 2026-06-05

**Authors:** Shekoufeh Forouzan, Sajjad Abbasi, Ali Akbar Moosavi, Majid Baghernejad, Sayyed Mahmoud Enjavinezhad, Andrew Turner

**Affiliations:** 1 Department of Soil Science, College of Agriculture, Shiraz University, Shiraz, Iran; 2 Department of Earth Sciences, School of Science, Shiraz University, Shiraz, Iran; 3 Géosciences Environnement Toulouse, CNRS, IRD, Université de Toulouse, Toulouse, France; 4 School of Geography, Earth and Environmental Sciences, University of Plymouth, Plymouth, United Kingdom; University of Limpopo, SOUTH AFRICA

## Abstract

Microplastics (MPs) in soils are becoming increasingly recognised as important terrestrial contaminants, yet their vertical distribution below the plough layer remains poorly understood. Here, we examined 76 genetic soil horizons from 27 profiles along four transects spanning managed and unmanaged land uses in Fars Province, southwest Iran, to depths of up to 140 cm. A total of 392 MPs were recovered from 71 of 76 samples, comprising 342 fibres (87.2%), 48 fragments or films and two spherules, with concentrations reaching about 320 particles kg^-1^ of dry soil. Raman analysis of 98 representative particles showed a dominance of polyamides, polyesters and polyolefins. MP abundance, size and polymer type were heterogeneous among sites and horizons, and only a few statistically significant relationships with soil properties were observed (notably inverse relationships with sand at Darab and silt at Sarvestan). MPs occurred throughout the profiles, including the deepest horizons, with no consistent size- or polymer-related fractionation with depth. These observations indicate sustained MP accumulation in soils and suggest that downward transport can occur under arid to semi-humid conditions through percolation, drying-cracking of the substrate and bioturbation. Because strong local point sources were not evident at most sites and fibres dominated the assemblage, atmospheric deposition is considered a major contributor at the soil surface, although additional source-specific data are needed to confirm this inference. The persistence and vertical mobility of MPs in Fars soils indicate potential risks to subterranean ecosystems and groundwater quality.

## 1. Introduction

Plastic pollution is now a global environmental issue, and microplastics (MPs; < 5 mm) have been documented in marine, freshwater, atmospheric and terrestrial compartments. Soils are increasingly recognised as a major environmental reservoir, although terrestrial systems remain less studied than aquatic ones [1–4]. Studies have also expanded from simple occurrence data to the ecological and human-health relevance of plastic particles and their associated additives or sorbed contaminants [5–7].

In soil systems, MPs may originate from atmospheric fallout, agricultural plastics, sewage sludge and compost application, wastewater irrigation, litter fragmentation, traffic-related emissions, and urban or industrial activities [4,8–10]. Once introduced, soils can function both as sinks and as secondary sources because particles may be retained, remobilised by erosion or re-entrained into the atmosphere [2,11]. MPs are also relevant to soil functioning because they can influence aggregation, porosity, water retention, nutrient cycling, enzyme activity, plant performance and the transport of co-occurring contaminants [2,4,5].

Vertical distribution and migration are key to understanding the long-term fate of MPs in soils. After deposition at the surface, particles may be redistributed by infiltrating water, preferential flow, bioturbation, root growth, tillage and wet-dry or shrink-swell cracking, while interactions of MPs with soil aggregates can further affect their retention and mobility [2,12–15]. Because these processes may operate simultaneously, soil depth profiles can reflect both source history and post-depositional transport.

Despite rapid growth of soil-MP studies in the recent literature, major knowledge gaps remain. Many field studies have focused on surface soils or shallow depth intervals, and relatively few have examined genetically defined horizons or evaluated whether MPs penetrate well below the plough layer [12,16]. Data are even scarcer for arid and semi-arid regions, where episodic rainfall, sparse vegetation, strong evaporative conditions, soil cracking and wind redistribution may influence both deposition and vertical transport differently from more humid environments [3,17]. As a result, the extent to which MPs are retained near the surface or migrate to deeper horizons in dryland soils remains insufficiently understood.

Against this background, the present study investigates the occurrence and vertical distribution of MPs in pedogenetic horizons from contrasting land uses and climatic settings in Fars Province, southwest Iran. Specifically, the objectives were to: (i) quantify MPs in genetic soil horizons to depths exceeding 1 m; (ii) characterise their shape, size, colour and polymer composition; and (iii) evaluate whether vertical distributions covary with selected soil physical and chemical properties. By providing horizon-resolved field data from an arid to semi-humid setting, this study aims to improve understanding of MP persistence, downward migration and potential exposure of deeper soil biota and groundwater resources.

## 2. Materials and methods

### 2.1. Study area

Fars Province is located in southwest Iran. It covers an area of about 120,000 km^2^ and is home to about 5 million people, with about a third of the population concentrated in the provincial capital city of Shiraz. Province-wide mean annual temperature is about 17 °C and mean annual precipitation is about 330 mm, but climatic gradients are pronounced because of strong topographic contrasts. The more mountainous northern and northwestern areas are cooler and wetter, with cold winters and mild summers, whereas the eastern and southeastern lowlands are warmer and drier, with hot summers and more limited moisture. Thus, the province encompasses a clear range of environmental conditions, including cooler uplands, warmer lowlands, variable topography and a range of managed and unmanaged land uses [18].

The agricultural sector of Fars plays a key role in the production and security of food for Iran and makes a significant contribution to the gross national product. It is also responsible for around 95% of total annual water consumption of about 10 billion m^3^ [18]. Important agricultural products across the province include wheat, corn, rice, dates, barley, figs, pomegranates, cotton, walnuts, pistachios, citrus fruits, sugar beets and tomatoes. Agriculture is the dominant regional anthropogenic activity relevant to land management but rural settlements, roads, gardens, groundwater abstraction, livestock grazing and dispersed local infrastructure are also present in the wider landscape.

### 2.2. Sampling

**Fig 1:**
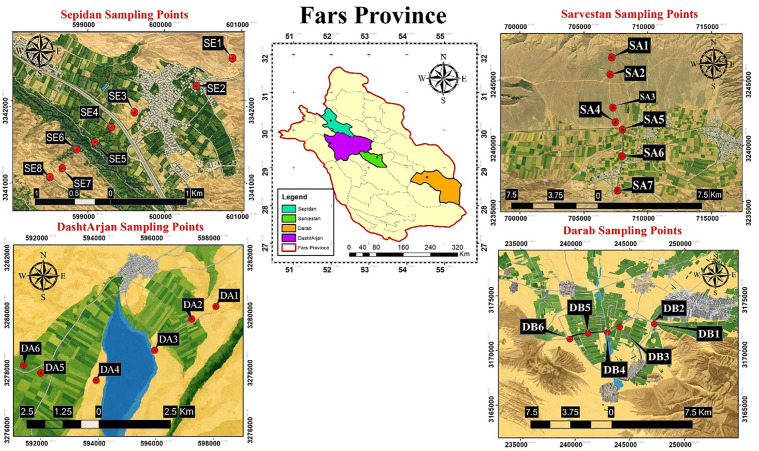
Location of the four transects and soil profiles sampled in Fars Province, southwest Iran. The map was drawn and designed by the authors as an original schematic land-use illustration and contains no satellite imagery or copyrighted basemap content..

Sampling was undertaken from the calcareous soils of the alluvial plains of four regions: Dasht Arjan, Darab, Sarvestan and Sepidan (Fig 1; Table 1); that encompassed different land uses. Specifically, agriculture for wheat and pistachios, second- and third-grade pastures (low vegetation cover), oak forest, private gardens and barren land subject to erosion and with some evidence of historical agricultural practices. Site selection prioritised settings without obvious intensive plastic inputs such as large greenhouse complexes, extensive contemporary plastic mulching or major industrial point sources.

**Table 1:** Land uses, profiles and genetic horizons sampled from the four regions of Fars Province under study. In parentheses are the lengths of each transect. For agriculture, the crops grown at the time of sampling are shown along with the source of irrigation water. H1-H4 denote successive genetic horizons from the topsoil downward; blank cells indicate that fewer than four distinct horizons were identified in that profile.

Region	Profile	Land use	H1, cm	H2, cm	H3, cm	H4, cm
Dasht Arjan	DA1	Pasture	0-25	25-70		
(7.2 km)	DA2	Agriculture (wheat, well-water)	0-30	30-65	65-100	
	DA3	Pasture	0-30	30-75	75-110	
	DA4	Pasture	0-25	25-50	50-90	
	DA5	Pasture	0-30	30-70	70-120	
	DA6	Forest	0-25	25-65	65-110	
Darab	DB1	Barren	0-25	25-60		
(8.0 km)	DB2	Barren	0-30	30-70	70-112	
	DB3	Pasture	0-30	30-75	75-115	
	DB4	Barren	0-30	30-95		
	DB5	Barren	0-30	30-70	70-140	
	DB6	Pasture	0-30	30-97		
Sarvestan	SA1	Barren	0-30	30-55		
(10.2 km)	SA2	Barren	0-14	14-75		
	SA3	Barren	0-45	45-110		
	SA4	Barren	0-35	35-75	75-130	
	SA5	Agriculture (pistachios, well-water)	0-30	30-80	80-140	
	SA6	Barren	0-25	25-60	60-95	
	SA7	Barren	0-15	15-45	45-80	80-140
Sepidan	SE1	Pasture	0-22	22-45	45-80	
(3.0 km)	SE2	Garden	0-15	15-35	35-65	
	SE3	Pasture	0-15	15-45	45-80	
	SE4	Pasture	0-15	15-40	40-65	
	SE5	Pasture	0-15	15-40	40-85	
	SE6	Garden	0-23	23-45	45-85	
	SE7	Agriculture (crops, rainfed)	0-20	20-60	60-100	100-130
	SE8	Pasture	0-25	25-60	60-110	

In each region, six to eight profiles were distributed along a sloping transect of about 3–10 km in order to capture topographic and land-use variability and allow comparisons among regions and horizons. Profiles were excavated with a stainless-steel shovel to the base of accessible genetic horizons, up to 1.4 m depth, so that MP occurrence could be evaluated well below the typical tillage depth and within pedogenic layers likely to integrate longer-term deposition and migration. Note that genetic horizons differ in thickness among profiles and that horizon-resolved results provide a pedological rather than uniformly depth-binned representation of vertical MP occurrence. Accordingly, interpretation of the results emphasises position within the profile and maximum depth reached rather than direct comparison of equal-thickness intervals.

Approximately 2 kg of soil (*n* = 76 in total), collected from each visible genetic horizon (two to four) with a smaller shovel, was stored in aluminium foil, transported to the laboratory, and passed through a 2-mm stainless-steel sieve.

### 2.3. Microplastic separation

After sieving and homogenisation, 50 g of each soil sample was transferred to a cleaned (washed in 50% HCl and rinsed with deionised water) 600 mL glass beaker using a stainless-steel spoon before the contents were loosely covered with foil and air-dried in a laminar flow hood (without airflow) for 24 h at 25 °C. The 50-g subsamples should be regarded as representative of the processed <2 mm fraction rather than that of the entire horizon mass.

In new (and cleaned) 600 mL glass beakers, organic matter was decomposed by oxidising 50 g of each sample in 300 mL of 30% H_2_O_2_ solution (Arman Sina, Tehran) at 25 °C until the cessation of bubble formation. The remaining material (and H_2_O_2_) was washed through a 150-mm diameter S&S filter paper (blue ribbon cellulose circle, grade 589/3, 2-μm pore size) with deionised water before residues, on their filters, were dried for 2 h in a sand bath at 60 °C and stored in individual glass Petri dishes.

Dried residues were added to saturated 300 mL solutions of ZnCl_2_ (Arman Sina, Tehran, in filtered deionized water; density ~ 1.7 g cm^-3^) in a series of cleaned 600 mL glass beakers. This solution permits recovery of both low-density polymers, such as polyethylene and polypropylene, and denser polymers, such as polyamides and polyethylene terephthalate. Following initial agitation at 350 rpm on a lateral shaker, the contents were allowed to settle for 24 h before the decanted contents were vacuum-filtered through S&S filter papers. This procedure was repeated twice, with the resulting filters being air-dried for 72 h at 25 °C in the laminar flow hood (without airflow) and transferred to individual Petri dishes. The extraction procedure has shown high overall recoveries (90.5 ± 1.44%), with polymer-specific recoveries of 95 ± 11.5% for PS, 77.77 ± 9.0% for PE, 80 ± 10.0% for PP, 88.88 ± 10.0% for PVC, and 93.84 ± 5.77% for ABS [19].

### 2.4. Microplastic identification and characterisation

Filters were inspected under a binocular microscope (Carl-Zeiss) at magnifications of up to 200 × , aided by ImageJ software, to identify, quantify and characterise microplastics. Criteria for identification included thickness, cross-sectional properties, shininess, hardness, surface structure and response to a hot, 250 μm diameter, stainless-steel probe. Potential natural fibres showing obvious tapering ends, cellular or lumen-like structures, twisted ribbon morphology or irregular pigmentation were excluded. Size measurements were based on the length along the longest axis (*L*), and particles were classified as follows: *L* ≤ 100 µm (with an effective visual detection limit of about 30 µm), 100 < *L* ≤ 500 µm, and *L* > 500 µm. Within constraints arising from weathering, staining and fading, colour was categorised as white-transparent, yellow-orange, red-pink, blue-green or black-grey, and shape was categorised as fibre (length: diameter ratio > 3), film-fragment or spherule-granule.

The polymeric makeup of 98 MPs, encompassing the observed range of shapes, sizes, colours, regions and depth intervals (specifically, from profiles DA5, DB1, DB3, DB4, DB5, SA1, SA3 and SE7), was determined using a micro-Raman spectrometer (µ-Raman-532-Ci, Avantes, Apeldoorn, the Netherlands) with a 785 nm laser and Raman shift window of 400−1800 cm^-1^. Polymer assignments were based on comparison of principal diagnostic bands with reference spectra and published band positions. Where spectra indicated more than one polymer, assignments were described conservatively as blended or mixed only when the main diagnostic bands of both materials were discernible. Heavily weathered, pigmented or very small particles may yield weaker or fluorescence-rich spectra, and these factors were considered when interpreting ambiguous results.

### 2.5. Minimisation and assessment of microplastic contamination

Measures were adopted to reduce airborne and procedural contamination, which is particularly important for fibrous MPs [20,21]. In the laboratory, windows and doors remained closed, benches were thoroughly cleaned using ethanol, and laboratory operators wore only cotton-based clothing to minimise shedding of synthetic fibres from garments. Glass, cellulose and stainless-steel materials were used wherever possible for sample contact, all reagents and solutions employed (including deionised water) were vacuum-filtered through 2 μm before use, and aluminium foil was used to cover equipment, beakers, filters and Petri dishes while not in use. Sample handling, drying and storage were performed in a laminar-flow hood with airflow switched off during exposure steps, and containers were opened only briefly during transfer, filtration and inspection. Samples were also processed individually rather than in open parallel batches.

Contamination in the laboratory was assessed by processing ten aliquots of filtered deionised water according to the protocols outlined above and treating them as procedural blanks. Microscopic analysis of these blanks revealed no detectable MPs and no blank correction was, therefore, applied to the soil data. While this result supports the effectiveness of the laboratory precautions, it does not substitute for dedicated field blanks, which remain desirable for fully evaluating potential contamination introduced during sampling and transport.

### 2.6. Physical and chemical properties of soils

In order to compare texture and geochemistry among horizons, profiles and regions, physical and chemical parameters were determined on the air-dried <2 mm soil fractions or on their saturated pastes/extracts using established techniques (e.g.,[22–24]). Briefly, organic carbon was measured by wet oxidation with potassium dichromate and sulphuric acid; texture was determined by hydrometry after dispersion with sodium hexametaphosphate; pH was measured directly in pastes and electrical conductivity measured in the corresponding saturation extracts; soluble CO_3_^2-^ and HCO_3_^-^ concentrations were determined by titration with sulphuric acid; calcium carbonate equivalent was determined by neutralisation with HCl; soluble Mg and Ca concentrations were determined by EDTA titration; and soluble Na and K concentrations were measured in the extracts using a Corning 405 flame photometer.

### 2.7. Data presentation and statistics

MP data are presented as both number per sample (50 g of < 2 mm soil) and concentration on a dry-weight basis (number per kg of < 2 mm soil). Data normality was assessed for each regional dataset using the Kolmogorov-Smirnov test, and parametric analyses were used when assumptions of normality were met and non-parametric tests were applied otherwise. Correlations between MP abundance and soil properties were evaluated region by region using Pearson’s or Spearman’s tests as appropriate, and, because of unequal group sizes and heterogeneous variance, differences among land-use categories were tested using the Kruskal-Wallis test. Because replication among some land-use categories was limited and MP distributions were highly heterogeneous, the statistical analysis is interpreted primarily as exploratory rather than fully predictive.

## 3. Results

### 3.1. Soil characteristics

The physical and chemical characteristics of the < 2 mm soils from each transect (region), profile and genetic horizon are summarised in Table 2, while the full analytical dataset is provided in S1 Table. Values of pH revealed that soils were either neutral or, mostly, alkaline (pH > 7.5), and exhibited little variation with transect distance, elevation or soil depth. Overall, pH wass lowest at Sepidan (mean = 7.37) and greatest at Darab (7.91). Electrical conductivity (EC) was variable between and within the regions but was always non-saline (< 2 dS m^-1^) or very slightly saline (< 4 dS m^-1^). Values were mostly between 0.30 and 0.60 dS m^-1^ at Sepidan (mean = 0.43 dS m^-1^), with a small but persistent decrease with soil depth. By contrast, at Dasht Arjan, Darab and Sarvestan EC ranged from 0.35 to 2.27 dS m^-1^ (mean = 1.05 dS m^-1^), 0.58 to 1.92 dS m^-1^ (mean = 1.16 dS m^-1^) and 0.50 to 2.75 dS m^-1^ (mean = 1.50 dS m^-1^), respectively, and there were no systematic trends with soil depth.

**Table 2:** A summary of the physical and chemical properties of the < 2 mm soil samples by region, profile and horizon (horizon data are separated by/). EC = electrical conductivity; OC = organic carbon.

Region	Profile	pH	EC, dS m^-1^	Sand, %	Clay, %	OC, %
Dasht Arjan	DA1	7.66/7.75	0.56/1.05	33.7/36.9	17.5/21.0	1.73/0.23
	DA2	7.72/7.81/7.84	0.70/1.75/0.63	44.1/45.8/44.1	13.9/19.2/15.7	1.13/0.54/0.89
	DA3	7.89/7.48/7.85	1.66/1.40/2.27	22.7/24.4/22.7	21.0/22.8/26.4	2.14/2.63/1.03
	DA4	7.83/8.06/7.80	0.35/0.61/1.22	24.4/33.4/36.9	24.6/21.0/19.2	1.26/0.21/0.15
	DA5	7.98/7.45/7.92	0.61/0.52/0.47	38.7/49.4/51.2	19.2/19.2/15.7	0.83/0.19/0.21
	DA6	7.91/8.08/8.02	0.79/2.62/0.65	31.6/28.5/32.1	21.0/28.1/31.7	1.01/0.44/0.42
Darab	DB1	7.80/7.90	0.89/0.86	28.0/28.0	22.8/33.5	1.03/0.64
	DB2	7.46/8.20/7.88	0.79/1.05/1.05	26.2/26.2/26.2	21.0/22.8/22.8	0.09/0.25/0.35
	DB3	7.63/7.75/7.93	1.66/1.92/1.48	38.4/20.3/16.3	23.9/38.0/38.0	1.06/0.54/0.45
	DB4	8.05/8.69	1.22/1.31	29.8/24.4	28.1/33.5	0.52/0.44
	DB5	7.60/7.96/7.96	0.50/1.40/1.13	40.5/29.8/28.0	15.7/31.7/29.9	0.54/0.15/0.35
	DB6	7.95/7.90	1.40/0.67	60.1/54.7	6.8/6.8	0.05/0.05
Sarvestan	SA1	7.17/7.49	1.01/0.58	41.9/29.7	27.5/39.7	1.29/0.37
	SA2	7.63/7.57	0.69/0.53	41.7/29.8	16.1/31.7	1.08/0.37
	SA3	7.43/7.45	0.50/1.11	39.6/31.8	23.9/33.7	0.70/0.01
	SA4	7.52/7.49/7.37	0.52/1.17/2.08	37.8/23.8/33.8	23.7/39.6/31.8	0.37/0.05/0.29
	SA5	7.52/7.57/7.48	0.90/1.56/2.24	47.7/51.8/47.8	17.8/13.7/15.6	0.80/0.01/0.11
	SA6	7.51/7.55/7.55	1.79/2.24/2.25	45.9/55.9/58.1	13.7/3.5/1.5	0.98/0.13/0.27
	SA7	7.58/7.54/7.59/7.60	1.77/2.75/2.55/2.25	51.7/58.1/66.0/68.0	6.5/1.4/1.9/1.5	0.64/0.02/0.01/0.01
Sepidan	SE1	7.30/7.37/7.36	0.47/0.43/0.40	23.0/19.5/23.0	27.8/36.7/36.7	2.53/0.58/0.58
	SE2	7.60/7.22/7.25	0.69/0.53/0.56	33.7/24.8/30.1	24.2/27.8/33.1	2.53/1.07/0.78
	SE3	7.34/7.37/7.39	0.47/0.39/0.41	24.8/21.2/28.4	27.8/36.7/33.1	1.65/0.87/0.87
	SE4	7.35/7.51/7.34	0.57/0.40/0.43	42.6/43.0/40.8	27.8/26.0/27.8	2.92/0.78/0.39
	SE5	7.28/7.30/7.40	0.46/0.35/0.33	33.7/30.1/26.6	22.4/27.8/34.9	1.17/0.39/0.39
	SE6	7.48/7.34/7.29	0.52/0.46/0.40	35.5/35.5/39.1	26.0/26.0/27.8	0.97/0.48/0.29
	SE7	7.39/7.32/7.37/7.45	0.40/0.30/0.32/0.26	31.9/23.0/30.1/35.5	26.0/27.8/31.4/29.6	0.48/0.19/0.25/0.05
	SE8	7.36/7.36/7.40	0.42/0.39/0.28	56.9/43.0/57.2	18.9/18.9/17.1	0.39/0.19/0.05

Organic carbon (OC) contents of the soils were variable between and within regions, with averages higher at Dasht Arjan and Sepidan (about 0.85%) than at Darab and Sarvestan (< 0.45%). Despite this variation, there was, in most cases, a reduction in OC with increasing soil depth. This reduction was most persistent at Sepidan and greatest, quantitatively, at Sarvestan, where values decreased by more than an order of magnitude in two profiles. Soil texture is summarised in terms of the percentages of sand and clay (with the remainder comprising silt). Thus, at Darab an increase in clay content and decrease in sand content was observed with increasing depth, while at Sepidan an increase in clay but similar sand content was generally found. At Dasht Arjan there was a similar distribution of clay and sand with depth, but at Sarvestan clay and sand distributions were more variable and a shift towards more sand and less clay was observed with increasing elevation along the transect.

### 3.2. Microplastic abundance and distributions

**Fig 2:**
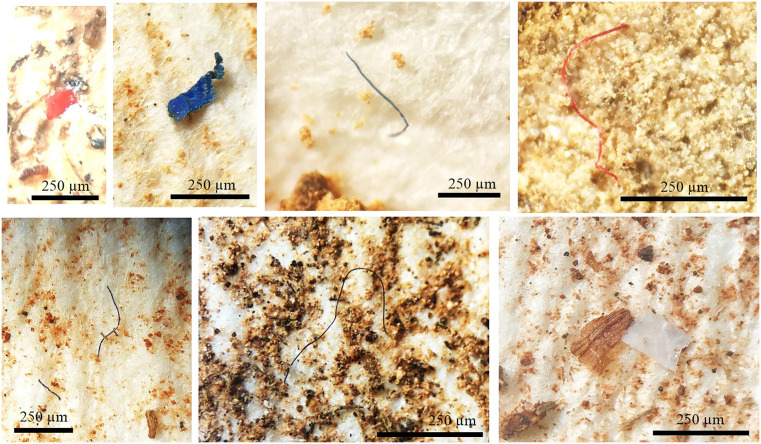
Examples of fibrous and sheet-like MPs isolated from the soils in the present study and identified under the microscope. All micrographs shown are original images acquired by the authors using a laboratory microscope and camera system.

MPs isolated from the soil samples are exemplified in Fig 2, and their horizon-resolved abundances are summarised in Table 3. In total, 392 MPs were retrieved, comprising 342 fibres, 48 fragments or (mainly) films, and two spherules. Overall, MPs were present in all but five soil samples and fibres were present in all but seven samples. Fibres appeared to be broadly distributed throughout horizons at the four locations, and were most abundant at Sepidan (134 in 25 soils) and least abundant at Sarvestan (45 in 19 soils). Other shapes were least abundant at Sarvestan (1 in 19 soils) but most abundant at Dasht Arjan (24 in 17 soils). As a consequence, the ratio of other shapes to fibres ranged from about 0.02 at Sarvestan to about 0.36 at Dasht Arjan. Regarding individual profiles, the highest numbers of fibres (> 20) were observed in DB2, DB3 and SA7 (barren land), DB3 and SE1 (pasture) and SE7 (rainfed agriculture). The highest numbers of other shapes were encountered at three sites in Dasht Arjan consisting of agriculture, pasture and forest.

**Table 3:** Number and size classification of microplastics (categorised as fibres and other) in 50-g (dry) < 2 mm soil samples by region, profile and horizon (horizon data are separated by/).

Region	Profile	Fibres	Other	<100 μm	>500 μm
Dasht Arjan	DA1	2/3	0/0	1/0	0/0
	DA2	1/1/5	0/1/6	0/1/5	0/0/0
	DA3	6/0/5	0/1/0	4/1/0	2/0/1
	DA4	5/5/4	0/0/1	1/2/4	2/0/0
	DA5	3/6/2	0/3/5	2/5/5	0/0/0
	DA6	10/6/3	1/1/5	1/0/4	0/1/0
	total	27/21/19 = 67	1/6/17 = 24	9/9/18 = 36	4/1/1 = 6
					
Darab	DB1	2/5	3/3	4/3	0/0
	DB2	14/9/11	0/1/0	2/5/2	1/1/0
	DB3	2/16/8	0/0/0	0/4/2	0/1/1
	DB4	2/2	0/0	0/0	0/0
	DB5	5/9/6	0/0/1	3/3/3	0/1/0
	DB6	3/2	1/2	1/4	0/0
	total	28/43/25 = 96	4/6/1 = 11	10/19/7 = 36	1/3/1 = 5
					
Sarvestan	SA1	4/3	0/0	0/2	0/0
	SA2	0/0	0/0	0/0	0/0
	SA3	1/3	0/0	0/0	1/0
	SA4	1/0/1	0/0/0	0/0	0/0
	SA5	4/1/2	0/1/0	0/0/1	1/2/0
	SA6	0/1/0	0/0/0	0/0/0	0/0/0
	SA7	4/3/7/10	0/0/0/0	2/0/5/2	1/0/0/1
	total	14/11/10/10 = 45	0/1/0/0 = 1	2/2/6/2 = 12	3/2/0/1 = 6
					
Sepidan	SE1	7/5/10	0/0/1	2/1/1	1/1/0
	SE2	8/4/4	3/2/1	1/2/0	1/0/0
	SE3	10/3/6	2/0/2	5/0/3	1/0/0
	SE4	2/1/4	1/0/0	1/0/1	0/0/0
	SE5	8/6/1	0/0/0	1/2/0	0/0/1
	SE6	2/0/7	0/1/0	0/1/3	0/0/0
	SE7	3/9/6/9	0/0/0/1	0/3/2/3	0/0/0/0
	SE8	8/5/6	0/0/0	1/0/1	1/1/0
	total	48/33/44/9 = 134	6/3/4/1 = 14	11/9/11/3 = 34	4/2/1/0 = 7

With respect to MP size, 118 particles were small (< 100 µm) and 24 were large (> 500 µm), with ratios of small to large ranging from 2 at Sarvestan to about 7.2 at Darab. Fibres exhibited a range of colours but were mainly black-grey or blue-green throughout. Non-fibrous particles also displayed various colours, but fragments and films from Darab, Sepidan and Sarvestan were dominated by black-grey or white-transparent particles, whereas Dasht Arjan contained proportionally more orange and red-pink particles.

Correlation analysis revealed few statistically significant relationships between MP abundance and soil characteristics. Specifically, at Darab, where a downward decrease in the ratio of sand to clay was observed, MPs were inversely correlated with the percentage of sand (*r*_s_ = −0.551, *p* = 0.033; *n* = 15) and positively correlated with soluble Na content (*r*_s_ = 0.592, *p* = 0.020; *n* = 15). At Sarvestan, where the ratio of sand to clay increased with elevation, MPs were inversely correlated with the percentage of silt (*r*_s_ = −0.563, *p* = 0.012; *n* = 19) and positively correlated with soluble K content (*r*_s_ = 0.465, *p* = 0.025; *n* = 19). No significant differences in MP concentrations were detected among land-use categories represented by more than two profiles (Kruskal-Wallis, *p* > 0.05).

**Fig 3:**
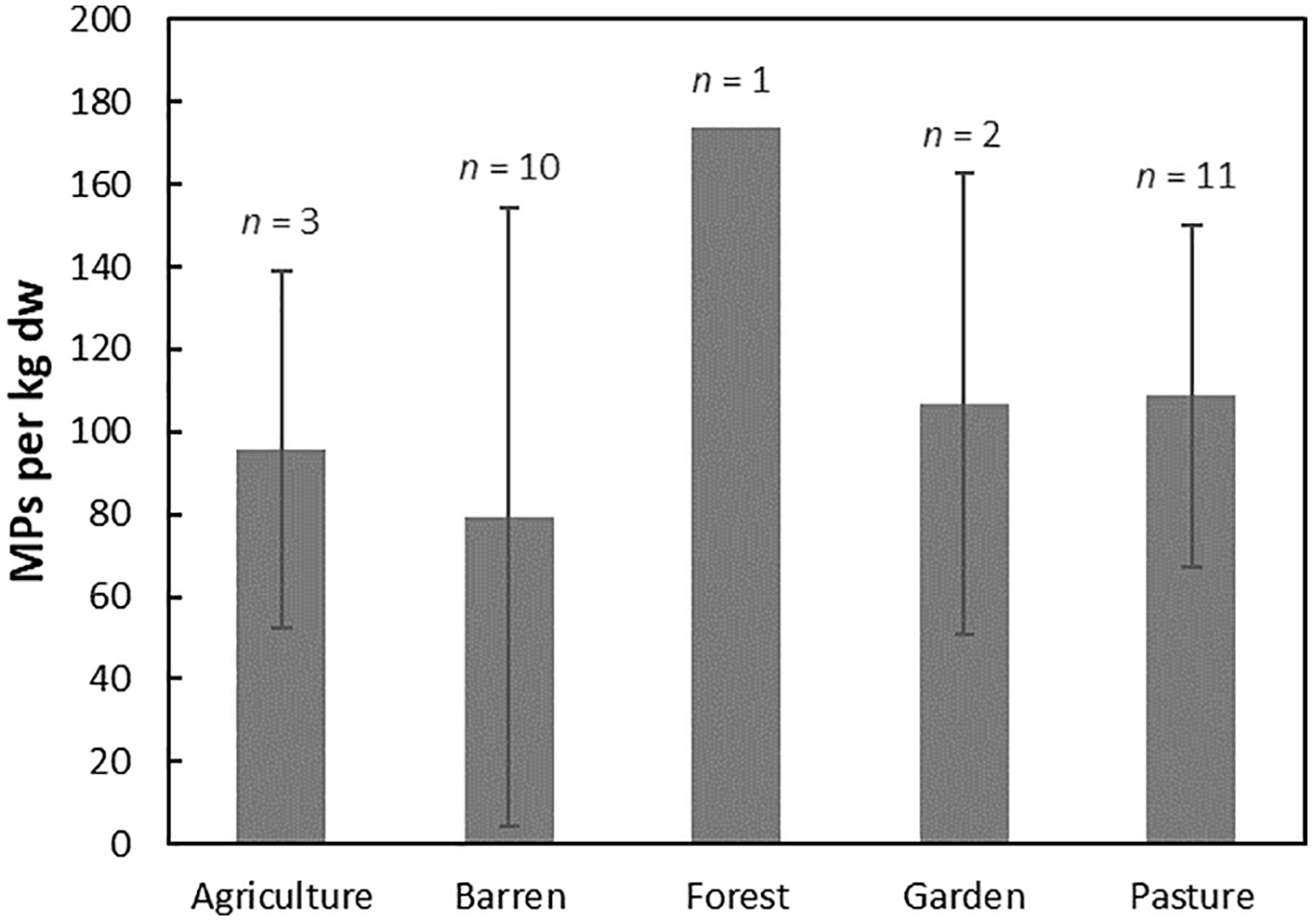
Mean and standard deviation of MP concentrations per kg of dry, < 2 mm soil, calculated from the summed number of particles per profile divided by the number of soils sampled in each profile (*n* is the number of profiles).

Fig 3 shows the mean concentrations of MPs in the soil samples, calculated from the summed number of particles in each horizon normalised to 1 kg and divided by the number of samples per profile, by land-use type. The highest mean concentration occurs in land-use categories represented by only one profile, while the error bars for the remaining categories highlight large within-class variability. This heterogeneity, together with uneven replication among land uses, limits strong inference about land-use effects and supports a cautious interpretation of the dataset.

### 3.3. Polymeric makeup of MPs

The results of μ-Raman analysis of suspected MPs from eight profiles encompassing all four regions are summarised in Table 4. Polyamide, mainly nylons, was the most widely distributed and abundant polymer group, although in a few cases nylon appeared to be blended with polypropylene. Polyester (including polyethylene terephthalate) and pure polypropylene were next in abundance but showed unequal distributions between the regions (for example, the latter was absent from Dasht Arjan and the former absent from Sepidan). Polyethylene was detected in each region and polystyrene was absent from Sarvestan. Among the other category were acrylonitrile butadiene styrene, ethylene-vinyl acetate, various polydienes and the biopolymer polylactic acid. At the level resolved here, polymers appeared to be heterogeneously distributed, with no clear evidence of systematic fractionation by density along transects or with profile depth.

**Table 4:** Distribution of polymers in 98 suspected MPs isolated from soil samples across the four regions.

Polymer	Dasht Arjan	Darab	Sepidan	Sarvestan	Total
Polyamide	5	24	12	4	45
Polyester	7	3	0	3	13
Polyethylene	2	3	2	1	8
Polypropylene	0	6	6	1	13
Polystyrene	1	2	1	0	4
Other or unidentified	1	10^a^	4	0	15
Total	16	48	25	9	98

^a^Includes one sample that was inorganic.

## 4. Discussion

The results of our study show that MPs are widespread throughout Fars soils at concentrations up to about 320 particles kg^-1^ dry soil, but that their abundance and characteristics are highly heterogeneous with respect to location, land use, horizon and soil properties. Comparable heterogeneity among soils and land-use settings has been reported elsewhere, although the strength and direction of associations are often site-specific [12,25–33]. In the present study, significant relationships with soil properties were limited to a small number of correlations in Darab and Sarvestan, suggesting that local textural and geochemical controls may influence retention or migration, but that no single soil property explains MP abundance across all regions.

The dominance of fibres, together with the absence of obvious strong point sources at most sites, is consistent with atmospheric deposition being an important contributor to surface-soil contamination in the study area. However, the present dataset does not exclude alternative or additional sources such as irrigation water, legacy agricultural inputs, local litter fragmentation, textile fibres or other diffuse anthropogenic inputs. Future source apportionment and confirmation of atmospheric deposition would require direct depositional measurements, deployment of field blanks and a comparison of polymer-shape between suspected sources.

A key result of the present investigation is the occurrence of MPs throughout the soil profiles, including horizons deeper than 1 m. Previous studies have reported MPs below the soil surface, but many were limited to arbitrary depth intervals of a few tens of centimetres rather than genetically defined horizons [12,16,27]. The current data, therefore, extend evidence for substantial vertical penetration of MPs in soils and suggest that deeper pedogenic layers may preserve a longer-term record of MP deposition and transport.

Several mechanisms may contribute to the downward movement of MPs once deposited at the soil surface. Percolating precipitation can transport particles through pore space; bioturbation and root activity can facilitate redistribution; and, in dryland or seasonally dry environments, shrink-swell behaviour and soil cracking may provide additional pathways [13–15]. In theory, smaller and denser particles may migrate more readily, yet the present data do not show clear systematic fractionation of size, shape or polymer type with depth. This suggests that multiple transport mechanisms probably operate together and that any sorting is minimal relative to the heterogeneity introduced by deposition and local soil structure.

The persistence of fibres and other MPs throughout the profiles has several environmental implications. In soils, MPs may alter structure, aeration, water movement, aggregation and plant performance, and their transfer into pore water may facilitate the co-transport of sorbed contaminants [2,5]. In arid to semi-humid regions, such as Fars Province, episodic rainfall following dry periods may favour both wet deposition and rapid infiltration into cracks, potentially enhancing vertical redistribution relative to more humid systems where near-surface retention may be stronger. The timescales recorded here are uncertain, but the presence of MPs in deep horizons is compatible with cumulative deposition and migration over years to decades rather than only very recent contamination. From a management perspective, mitigation should focus on reducing diffuse plastic emissions to soil and air, improving management of synthetic textiles and agricultural plastics, controlling contaminated irrigation inputs and extending routine monitoring beyond surface soils.

### 4.1. Study limitations

This study has several limitations that should be considered when interpreting the dataset. First, particles smaller than about 30 µm were not quantified, meaning that the total burden of small MPs is likely underestimated. Second, although procedural laboratory blanks were analysed and no MPs were detected, dedicated field blanks were not available to fully evaluate contamination during sampling and transport. Third, polymer identification was performed on a representative subset rather than on all particles retrieved, and quantitative recovery experiments were not conducted specifically for the present soil dataset. Finally, because direct source measurements were not available, the role of atmospheric deposition is inferred from the dominance of fibres and the lack of obvious point sources rather than demonstrated through source apportionment. These limitations do not alter the evidence for widespread and deep-soil occurrence, but mean that source attribution and total particle burdens should be interpreted with some caution.

## 5. Conclusions

This study demonstrates that MPs, dominated by fibres and reaching concentrations of about 320 particles kg^-1^, are widely and heterogeneously distributed in soils from four contrasting regions of Fars Province, Iran. A major strength of the work is the use of genetically defined horizons across contrasting land uses and climatic settings, allowing deep-soil occurrence to 140 cm to be evaluated beyond the shallow intervals that dominate much of the current literature. Despite the omission of particles smaller than about 30 µm, the absence of field blanks, and the lack of direct source measurements, the consistent presence of MPs at depth indicates substantial downward migration from the soil surface over time. Atmospheric deposition is likely an important contributor to this surface loading, although additional source-specific measurements are needed to confirm its relative importance. The results imply potential exposure of subterranean biota and, in the longer term, groundwater resources, and highlight the need for future soil MP studies to extend beyond surface horizons and to better understand both transport pathways and source apportionment.

## Supporting information

S1 Table Full analytical dataset of soil physical and chemical properties and microplastic abundance and characteristics by region, profile and genetic horizon. (XLSX)

S1 Text Completed PLOS questionnaire on inclusivity in global research. (DOCX)

S1 Fig Graphical_Abstract. (PNG)
