# Supplementary material for: Vertical distribution and migration of microplastics in soils from Fars Province, Southwest Iran
Source: PLoS One. 2026 Jun 5;21(6):e0333572. doi: 10.1371/journal.pone.0333572 (PMC13241006; doi:10.1371/journal.pone.0333572)
Supplement: S1 Text — (DOCX) [file pone.0333572.s002.docx]

**Inclusivity in global research questionnaire**

**Manuscript:** Vertical distribution and migration of microplastics in soils from Fars Province, southwest Iran

**Journal:** PLOS ONE

# Ethical considerations, permits and authorship

**Question:** Provide details as to who granted permissions and/or consent for the study to take place and whether there were local collaborators.

**Response:** This study involved collection and analysis of soil samples from Fars Province, Iran. The study was conducted with substantial local collaboration, and the author team includes researchers based at Shiraz University in Iran, where the field sampling was organized and carried out. The work did not involve human participants, Indigenous populations, cultural artefacts, or protected biological specimens. Sampling focused on soils from non-protected land-use settings described in the manuscript. To the best of the authors’ knowledge, no special community consent procedures were required for this non-human environmental sampling; site access and field activities were arranged locally by the Iranian research team.

**Question:** How were local researchers and institutions included in authorship and study leadership?

**Response:** Local researchers and institutions were central to the study design, field sampling, laboratory work, interpretation, and manuscript preparation. Shiraz University researchers are included as authors, and the study area, sampling design, and local environmental interpretation were developed with direct involvement of Iran-based coauthors. This authorship structure reflects substantive scientific contributions rather than courtesy inclusion.

# Human subjects research

**Question:** Did the study involve human participants, community consent procedures, or informed consent documents?

**Response:** Not applicable. This study did not involve human participants, interviews, surveys, medical data, or community-based human subjects research.

**Question:** How did local community members provide input on the aims, methodology, or outcomes?

**Response:** Not applicable for human subjects research. However, the environmental context and field implementation were informed by local scientific expertise through the Iran-based coauthors and institutions involved in the work.

**Question:** Will findings be made available in an understandable format to stakeholders in the study region?

**Response:** Yes. The findings will be disseminated through the publication, author communications, and the participating institutions, including local academic stakeholders in Iran.

# Non-human subjects research

**Question:** Did the permission obtained from local authorities include agreements on access to outputs and benefit sharing?

**Response:** The study was conducted through collaboration with local academic researchers and institutions in Iran, and outputs are shared through coauthorship and scholarly dissemination. Because this work involved non-human environmental soil samples rather than regulated biological or heritage materials, formal benefit-sharing agreements of the type used for biological or cultural collections were not applicable.

**Question:** If the material used in the study was imported, provide the year of import and import/export permits.

**Response:** Not applicable. The soil samples analyzed in this study were collected in Iran for the purposes of this study and were not imported as archival or transferred specimens.

**Question:** If archival specimens were used, state how the material was acquired and any permits obtained.

**Response:** Not applicable. The study did not use archival specimens or museum/collection materials.

**Question:** How was the potential cultural significance of the materials considered in the research design?

**Response:** The study focused on environmental soil samples from land-use transects and did not involve culturally sensitive materials, human remains, Indigenous heritage, or archaeological resources. Accordingly, no specific cultural-significance concerns were identified for the sampled material.

**Question:** If the manuscript includes photographs of human remains, indicate whether permission was obtained.

**Response:** Not applicable. The manuscript does not include photographs of human remains.

**Author note:** This questionnaire is provided in response to the journal’s request for additional transparency regarding inclusivity in global research. The study was conducted with direct involvement of local researchers and institutions in the country where the fieldwork took place.
