# Supplementary figures and images for: Vertical distribution and migration of microplastics in soils from Fars Province, Southwest Iran
Source: PLoS One. 2026 Jun 5;21(6):e0333572. doi: 10.1371/journal.pone.0333572 (PMC13241006; doi:10.1371/journal.pone.0333572)

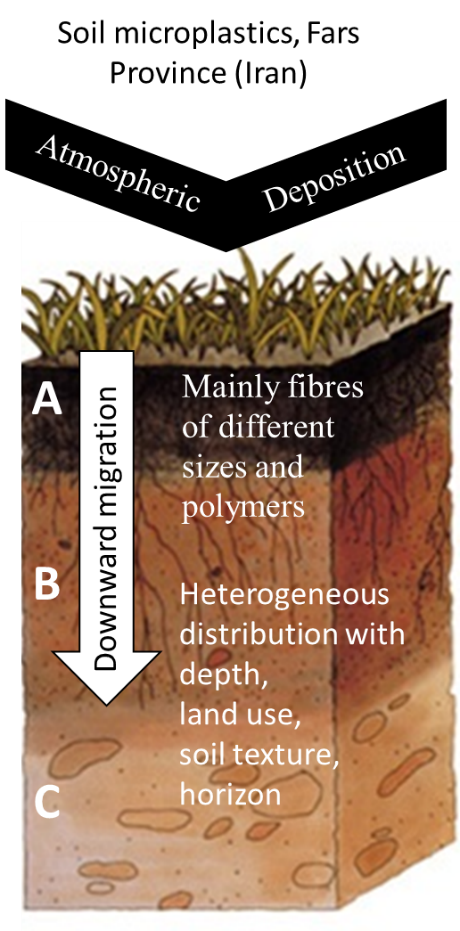

Supplement: S1 Fig — (PNG) [file pone.0333572.s003.png]
